# Integration of Urinary EN2 Protein & Cell-Free RNA Data in the Development of a Multivariable Risk Model for the Detection of Prostate Cancer Prior to Biopsy

**DOI:** 10.3390/cancers13092102

**Published:** 2021-04-27

**Authors:** Shea P. Connell, Robert Mills, Hardev Pandha, Richard Morgan, Colin S. Cooper, Jeremy Clark, Daniel S. Brewer, The Movember GAP1 Urine Biomarker Consortium

**Affiliations:** 1 Norwich Medical School, University of East Anglia, Norwich Research Park, Norwich NR4 7TJ, UK; sheaconnell@gmail.com (S.P.C.); colin.cooper@uea.ac.uk (C.S.C.); Jeremy.Clark@uea.ac.uk (J.C.); 2 Norfolk and Norwich University Hospitals NHS Foundation Trust, Norwich, Norfolk NR4 7UY, UK; robert.mills@nnuh.nhs.uk; 3 Faculty of Health and Medical Sciences, The University of Surrey, Guildford GU2 7XH, UK; h.pandha@surrey.ac.uk; 4 School of Pharmacy and Medical Sciences, University of Bradford, Bradford BD7 1DP, UK; r.morgan3@bradford.ac.uk; 5 The Earlham Institute, Norwich Research Park, Norwich, Norfolk NR4 7UZ, UK

**Keywords:** prostate cancer, biomarker, urine, machine learning, TRIPOD, liquid biopsy

## Abstract

**Simple Summary:**

Prostate cancer is a disease responsible for a large proportion of all male cancer deaths but there is a high chance that a patient will die with the disease rather than from. Therefore, there is a desperate need for improvements in diagnosing and predicting outcomes for prostate cancer patients to minimise overdiagnosis and overtreatment whilst appropriately treating men with aggressive disease, especially if this can be done without taking an invasive biopsy. In this work we develop a test that predicts whether a patient has prostate cancer and how aggressive the disease is from a urine sample. This model combines the measurement of a protein-marker called EN2 and the levels of 10 genes measured in urine and proves that integration of information from multiple, non-invasive biomarker sources has the potential to greatly improve how patients with a clinical suspicion of prostate cancer are risk-assessed prior to an invasive biopsy.

**Abstract:**

The objective is to develop a multivariable risk model for the non-invasive detection of prostate cancer prior to biopsy by integrating information from clinically available parameters, Engrailed-2 (EN2) whole-urine protein levels and data from urinary cell-free RNA. Post-digital-rectal examination urine samples collected as part of the Movember Global Action Plan 1 study which has been analysed for both cell-free-RNA and EN2 protein levels were chosen to be integrated with clinical parameters (*n* = 207). A previously described robust feature selection framework incorporating bootstrap resampling and permutation was applied to the data to generate an optimal feature set for use in Random Forest models for prediction. The fully integrated model was named ExoGrail, and the out-of-bag predictions were used to evaluate the diagnostic potential of the risk model. ExoGrail risk (range 0–1) was able to determine the outcome of an initial trans-rectal ultrasound guided (TRUS) biopsy more accurately than clinical standards of care, predicting the presence of any cancer with an area under the receiver operator curve (AUC) = 0.89 (95% confidence interval(CI): 0.85–0.94), and discriminating more aggressive Gleason ≥ 3 + 4 disease returning an AUC = 0.84 (95% CI: 0.78–0.89). The likelihood of more aggressive disease being detected significantly increased as ExoGrail risk score increased (Odds Ratio (OR) = 2.21 per 0.1 ExoGrail increase, 95% CI: 1.91–2.59). Decision curve analysis of the net benefit of ExoGrail showed the potential to reduce the numbers of unnecessary biopsies by 35% when compared to current standards of care. Integration of information from multiple, non-invasive biomarker sources has the potential to greatly improve how patients with a clinical suspicion of prostate cancer are risk-assessed prior to an invasive biopsy.

## 1. Introduction

Prostate cancer is responsible for 13% of all male cancer deaths in the UK, yet this is contrasted by 10-year survival rates approaching 84% [1]. This dichotomy has led to uncertainty for clinicians in how best to diagnose and predict the outcome for prostate cancer patients to minimise overdiagnosis and overtreatment whilst appropriately treating men with aggressive disease [2]. More accurate discrimination of disease state in biopsy naïve men would mark a significant development compared to current standards and impact large numbers of patients suspected of harbouring prostate cancer. The development of such a pre-biopsy screening test would provide a convenient checkpoint along the clinical pathway for patients to exit without the need for further invasive and stressful follow-up.

Under current guidelines patients are selected for further clinical investigations for prostate cancer if they have an elevated prostate specific antigen (PSA) (≥4 ng/mL) and/or an adverse finding on digital rectal examination (DRE) or lower urinary tract symptoms, whilst other factors such as age and ethnicity are also considered alongside patient preference [3,4,5]. More recently multiparametric MRI (mpMRI) has been used as a triage tool to reduce negative biopsy rates since its validation in the PROMIS clinical trial [6]. However, as it has gained more widespread adoption, mpMRI has shown a higher rate of inter-operator and inter-machine variability than reported in controlled clinical trials; up to 28% of clinically significant disease is missed in practice [5,7,8,9]. Coupled with the relative expense, time and expertise required to undertake an mpMRI meeting the current clinical guidelines, there is a need to improve on current clinical practices.

Biomarkers utilising tissue samples taken at the time of diagnosis for the detection of aggressive or significant prostate cancer requiring clinical attention are relatively plentiful [10,11,12,13]. Many of these markers are good tests, whether that be for discerning the most aggressive disease [11,14], or for predicting disease-free survival following radical prostatectomy [15]. However, requiring tissue means a biopsy must already have been performed, making these tests incompatible with reducing the rates of unnecessary biopsy that come at considerable economic, psychological and societal cost to patients and healthcare systems alike [2,16,17].

As a secretory organ directly interacting with the male urinary tract, the prostate is well-placed as a candidate for non-invasive liquid biopsy from urine samples [18]. Single- or few-biomarker panels such as Engrailed-2 (EN2) protein expression [19], the SelectMDx [20] and ExoDx Prostate (IntelliScore) [21] tests have published promising results for the non-invasive detection of significant disease (Gleason score (Gs) ≥ 7). However, they are in various stages of clinical validation and none are currently implemented in the UK healthcare system [5]. Most urinary biomarkers developed to date for the prediction of biopsy outcome are unimodal; considering a singular fraction of urine (such as the cell-pellet or cell-free fractions) or biological aspect of cancer to appraise disease status. Whilst these tests have shown promising clinical use and accuracy, for the majority it has not yet been explored whether extra predictive value could be derived by integrating multiple streams of information from other sources.

Since initial development, the SelectMDx model has been updated to include clinically available parameters of serum PSA, patient age and DRE alongside urinary *HOXC6* and *DLX1* mRNA, adding significant predictive ability for patients with a PSA < 10 ng/mL [22]. We have also recently shown the benefit of such a holistic approach, presenting the development of the multivariable ExoMeth risk prediction model integrating clinical parameters, hypermethylation within the urinary cell pellet and urinary cell-free RNA expression data that displayed improved clinical utility over any single mode [23].

EN2 is a homeodomain-containing transcription factor that has an essential function in early development, which in mammals includes the delineation of the midbrain/hindbrain border [24]. For a transcription factor it has a number of unusual properties, including the ability to be secreted from cells and taken up by others [25]. Indeed, a recent study indicated that prostate cancer cells can secrete EN2 protein through vesicles which are then taken up by other non-EN2 expressing cells, where it can directly influence the transcription of target genes [25].

This secretory behaviour of EN2 makes it a potential biomarker for prostate cancer, and indeed EN2 protein can be detected in the urine of men with prostate tumours [19]. The original and subsequent studies have generally supported a diagnostic role for urinary EN2, including a relationship between urinary EN2 concentration and tumour volume [19,26]. More recently, a lateral flow-based test for EN2 has been described that could potentially allow point-of-care testing [27].

In this study, we report the utility of a predictive model produced by the integration of clinically available parameters, urinary EN2 protein levels and targeted cell-free RNA transcriptomics. The data were collected within the Movember Global Action Plan 1 (GAP1) study that explored a range of biomarkers in urine for PCa diagnosis and prognosis. The clinical utility of this model is determined by the ability to predict the presence of Gs ≥ 7 and Gs ≥ 4 + 3 disease on biopsy, both critical distinctions in clinical settings, where patients with Gs ≥ 7 are recommended radical therapy [5], whilst patients with Gs 4 + 3 have significantly worse outcomes than Gs 3 + 4 patients [28]. Aware that most cancer biomarkers and predictive models fail to reach clinical adoption, we have adhered to the guidelines for the transparent reporting of a multivariable prediction model for individual prognosis or diagnosis (TRIPOD) whilst developing the models and results presented here [29].

## 2. Materials and Methods

### 2.1. Patient Population and Characteristics

The full Movember GAP1 urine cohort comprises 1257 first-catch post-DRE urine samples collected between 2009 and 2015 from urology clinics at multiple sites, as described in Connell et al. (2019). As a diverse range of techniques was applied to samples from this cohort and restricted amounts of urine, the number of experiments that could be performed on any one sample was limited. Samples within the Movember cohort that were quantified for both EN2 levels by ELISA and cell-free-RNA (cf-RNA) expression by NanoString (Seattle, WA, USA) were eligible for selection for model development in the current study (*n* = 218).

Exclusion criteria for model development included a recent prostate biopsy or trans-urethral resection of the prostate (<6 weeks) and metastatic disease (confirmed by a positive bone-scan or PSA > 100 ng/mL), resulting in a cohort of 207 samples, deemed the ExoGrail cohort (Table 1). All samples analysed in the ExoGrail cohort were collected from the Norfolk and Norwich University Hospital (NNUH, Norwich, UK). Sample collections and processing were ethically approved by the East of England REC.

### 2.2. Sample Processing and Analysis

Urine samples were processed according to the Movember GAP1 standard operating procedure (Supplementary Methods). In brief, within 30 min of collection, urine was centrifuged (1200× *g* 10 min, 6 °C) to remove cellular material. Supernatant extracellular vesicles were harvested by microfiltration and cell-free mRNA extracted (RNeasy micro kit, #74004, Qiagen, Hilden, Germany) on the same day that they were provided by the patient. RNA was amplified as cDNA with an Ovation PicoSL WTA system V2 (Nugen, Redwood City, CA, USA, #3312-48). Urinary EN2 protein concentration was quantified by ELISA from whole urine using a monoclonal anti-mouse EN2 antibody, as described by Morgan et al. (2011) [19]. Cell-free mRNA was quantified from urinary extracellular vesicles using NanoString technology, with 167 gene-probes (Table S1), as described in Connell et al. (2019), with the modification that NanoString data were normalised according to NanoString guidelines using NanoString internal positive controls, and log_2_ transformed. Clinical variables serum PSA, age at sample collection, DRE finding, and urine volume collected were considered.

### 2.3. Statistical Analysis

All analyses, model construction and data preparation were undertaken in R version 3.5.3 [30], and unless otherwise stated, utilised base R and default parameters. All data and code required to reproduce these analyses can be found at the UEA Cancer Genetic GitHub repository [31].

### 2.4. Feature Selection

In total, 172 variables were available for prediction (cf-RNA (*n* = 167), clinical variables (*n* = 4) and urinary EN2 (*n* = 1); for full list see Table S1), making feature selection a key task for minimising model overfitting and increasing the robustness of trained models. To avoid dataset-specific features being positively selected [32], we implemented a robust feature selection workflow utilising the Boruta algorithm [33] and bootstrap resampling. Boruta is a random forest-based algorithm that iteratively compares feature importance against random predictors, deemed “shadow features.” Features that perform significantly worse compared to the maximally performing shadow feature at each permutation, (*p* ≤ 0.01, calculated by Z-score difference in mean accuracy decrease) are consecutively dropped until only confirmed, stable features remain.

Boruta was applied on 1000 datasets generated by resampling with replacement. Features were only positively selected for model construction when confirmed as stable features in ≥90% of resampled Boruta runs.

### 2.5. Comparator Models

To evaluate potential clinical utility, additional models were trained as comparators using subsets of the available variables across the patient population: a clinical standard of care (SoC) model was trained by incorporating age, PSA, T-staging and clinician DRE impression; a model using only the values from the EN2 ELISA (EN2, *n* = 1); and a model only using NanoString gene-probe information (NanoString, *n* = 167). The fully integrated ExoGrail model was trained by incorporating information from all of the above variables (*n* = 177). Each set of variables for comparator models were independently selected via the bootstrapped Boruta feature selection process described above to select the most optimal subset of variables possible for each predictive model.

### 2.6. Model Construction

All models were trained via the random forest algorithm [34], using the *randomForest* package [35] with default parameters except for resampling without replacement and 401 trees being grown per model. Risk scores from trained models are presented as the out-of-bag predictions; the aggregated outputs from decision trees within the forest where the sample in question has not been included within the resampled dataset [34]. Bootstrap resamples were identical for feature selection and model training for all models and used the same seed for random number generator.

Models were trained on a modified continuous label, based on biopsy outcome and constructed as follows: samples were scored on a continuous scale (range: 0–1) according to the dominant Gleason pattern: where 0 represented no evidence of cancer, Gleason scores 6 & 3 + 4 were assigned to 0.5 and Gleason scores ≥ 4 + 3 are set to 1. Following this categorisation, the score is treated as a continuous variable by the Random Forest algorithm described above. This process was designed to recognise that two patients with the same TRUS-biopsy Gleason score will not share the exact same proportions of tumour pattern, or overall disease burden. This scale was solely used for model training and was not represented in any endpoint measurements, or for determining the predictive ability and clinical utility.

### 2.7. Statistical Evaluation of Models

Area Under the Receiver-Operator Characteristic curve (AUC) metrics were produced using the *pROC* package [36], with confidence intervals calculated via 1000 stratified bootstrap resamples. Density plots of model risk scores, and all other plots were created using the *ggplot2* package [37]. Partial dependency plots were calculated using the *pdp* package [38]. Cumming estimation plots and calculations were produced using the *dabestr* package [39] and 1000 bootstrap resamples were used to visualise robust effect size estimates of model predictions.

Decision curve analysis (DCA) [40] examined the potential net benefit of using PUR-signatures in the clinic. Standardised net benefit (sNB) was calculated with the *rmda* package [41] and presented throughout our decision curve analyses as it is a more directly interpretable metric compared to net benefit [42]. In order to ensure DCA was representative of a more general population, the prevalence of Gleason scores within the ExoGrail cohort were adjusted via bootstrap resampling to match those observed in a population of 219,439 men that were in the control arm of the Cluster Randomised Trial of PSA Testing for Prostate Cancer (CAP) Trial [43], as described in Connell et al. (2019). Briefly, of the biopsied men within this CAP cohort, 23.6% were Gs 6, 8.7% Gs 7 and 7.1% Gs ≥ 8, with 60.6% of biopsies showing no evidence of cancer. These ratios were used to perform stratified bootstrap sampling with a replacement of the Movember cohort to produce a “new” dataset of 197 samples with risk scores from each comparator model. sNB was then calculated for this resampled dataset, and the process repeated for a total of 1000 resamples with replacement. The mean sNB for each risk score and the “treat-all” options over all of the iterations were used to produce the presented figures to account for variance in resampling. Net reduction in biopsies, based on the adoption of models versus the default treatment option of undertaking biopsy in all men with PSA ≥ 4 ng/mL was calculated as:

(1) $$Biopsy_{NetReduction}=\left(NB_{Model}-NB_{All}\right)\times \frac{1-Threshold}{Threshold}$$

where the decision threshold (*Threshold*) is determined by accepted patient/clinician risk [40]. For example, a clinician may accept up to a 25% perceived risk of cancer before recommending biopsy to a patient, equating to a decision threshold of 0.25.

## 3. Results

### 3.1. The ExoGrail Development Cohort

Urinary EN2 protein and cf-RNA data were available for 207 patients within the Movember GAP1 cohort, with all samples originating from the NNUH to form the ExoGrail development cohort (Table 1). The proportion of Gleason ≥ 7 disease in the ExoGrail cohort was 48%, whilst 25 patients were deemed to have no evidence of cancer (NEC, PSA < 4 ng/mL), and did not receive a biopsy.

### 3.2. Feature Selection and Model Development

**Figure 1:**
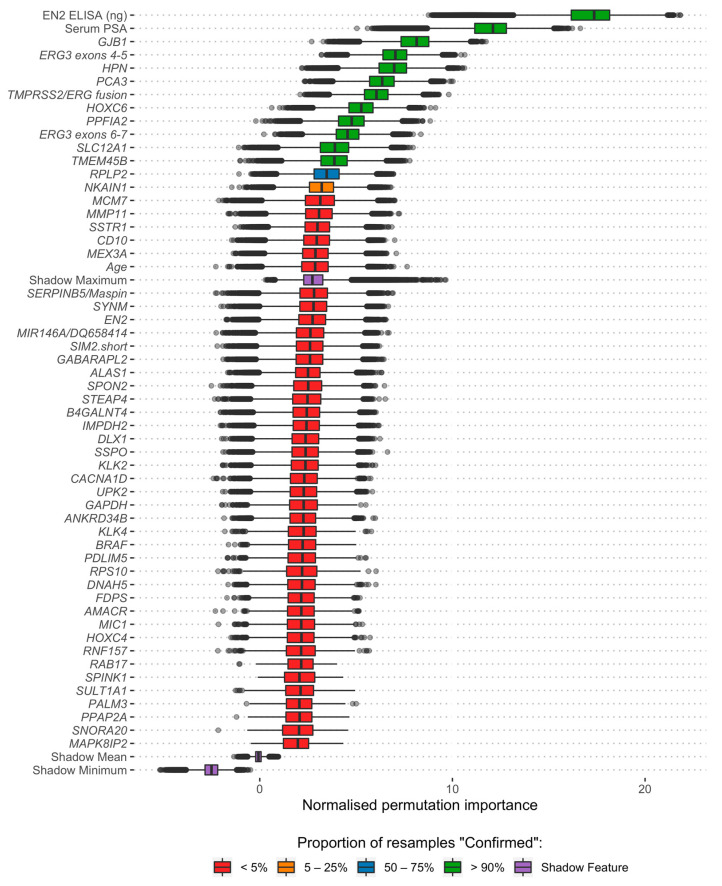
Analysis of variables available for the training of the ExoGrail model through the application of the Boruta algorithm via bootstrap resampling. 1000 resamples with replacement of the available data were made, with the normalised permutation importance of each variable recorded at each iteration, along with the decision of Boruta within that resample. Fill colour shows the proportion of resamples that a feature was positively retained by Boruta. Those features selected in ≥90% of resamples were selected for fitting predictive models. Variables rejected in all of the 1000 resamples are not shown here but are fully detailed in Table S1.

Using the robust feature selection framework, four models were produced in total: a standard of care (SoC) model incorporating only clinically available parameters (age and PSA), a model using urinary EN2 protein levels as the sole predictor variable (Engrailed), a model using only cf-RNA information (ExoRNA, 11 gene-probes) and the integrated model, named ExoGrail that incorporated variables from all three sources (12 variables) (Table 2). The ExoGrail model is a multivariable risk prediction model incorporating clinical parameters, urinary EN2 protein levels and cf-RNA expression information. When the resampling strategy was applied for feature reduction using Boruta, 12 variables were selected for the ExoGrail model. Each of the retained variables were positively selected in every resample and notably included information from clinical and cf-RNA variables, as well as urinary EN2 (Figure 1; full resample-derived Boruta variable importance for the SoC, Engrailed and ExoRNA comparator models can be seen in Figures S1–S3, respectively).

In the SoC comparator model, only PSA and age were selected as important predictors. Urinary EN2 levels were confirmed as important in the independent Engrailed model as the sole variable, and also within the ExoGrail model (Table 2). For the cf-RNA model, ExoRNA, 11 transcripts were selected, notably including both variants of the ERG gene-probe and *TMPRSS2/ERG* fusion gene-probe. ExoGrail incorporated an additional cf-RNA transctript, *SLC12A1*, which was not previously selected in the ExoRNA comparator model. When this was examined by partial dependency plots, an additive interaction effect was observed between quantified levels of urinary EN2 and counts of SLC12A1 on the predicted ExoGrail risk signature output (Figure S4).

### 3.3. ExoGrail Predictive Ability

**Figure 2:**
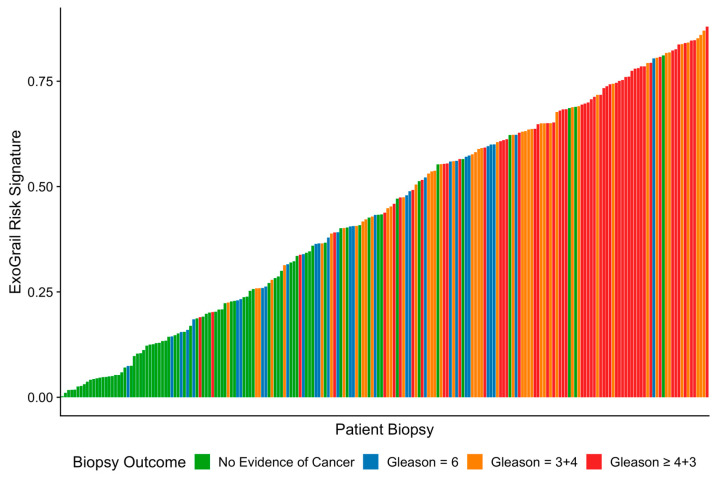
Representation of the ExoGrail risk score for each patient within a waterfall plot, where each coloured bar represents an individual’s biopsy outcome (fill colour) and predicted ExoGrail risk score (bar height). Green—No evidence of cancer, Blue—Gs 6, Orange—Gs 3 + 4, Red—Gs ≥ 4 + 3.

As ExoGrail Risk Score (range 0–1) increased, the likelihood of high-grade disease detection on TRUS-biopsy was significantly greater (Proportional odds ratio = 2.21 per 0.1 ExoGrail increase, 95% CI: 1.91–2.59; ordinal logistic regression, Figure 2). The median ExoGrail risk score for metastatic patients was 0.76 (*n* = 11). These patients were excluded from model training and can be considered as a positive control for model calibration.

ExoGrail was superior to all other models for the detection of Gleason ≥ 3 + 4 (AUC = 0.90 (95% CI: 0.86–0.94), *p* < 0.001, bootstrap test with 1000 resamples) and for any cancer (AUC = 0.89 (95% CI: 0.85–0.94), *p* < 0.001, bootstrap test with 1000 resamples) (Table 3). When Gleason ≥ 4 + 3 was considered, ExoGrail returned an AUC = 0.84 (95% CI: 0.78–0.89), outperforming the SoC and cf-RNA models (*p* < 0.001, bootstrap test with 1000 resamples), whilst the Engrailed model displayed similar performance by AUC metrics (Table 3). A model consisting of the combination of EN2 and PSA showed a similar ability in the detection of Gleason ≥ 4 + 3 compared to ExoGrail (AUCs of 0.85 compared to 0.84), whilst ExoGrail showed a small improvement in the detection of Gleason ≥ 3 + 4 disease and any cancer (Table S2).

**Figure 3:**
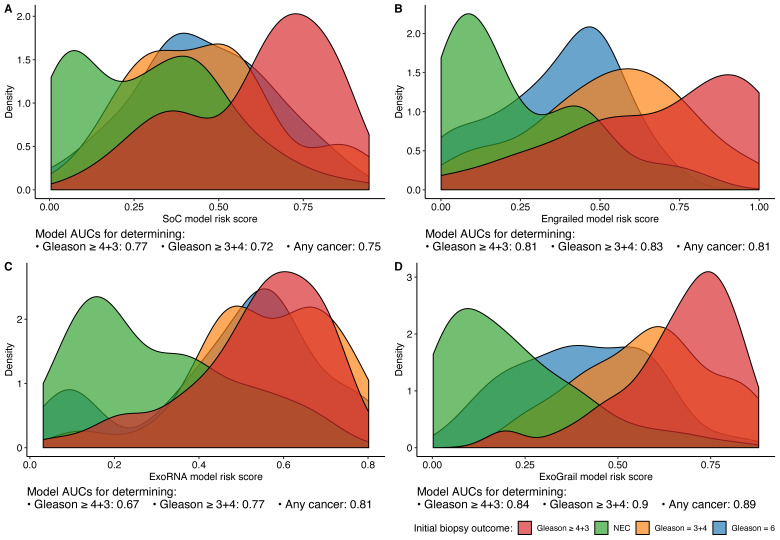
Risk score distributions of the four trained models, calculated as the out-of-bag predictions and represented as density plots. Area under the receiver operator curves (AUCs) for each model’s predictive ability for clinically relevant outcomes are detailed underneath each panel. Each random forest model was fit using different input variables; (**A**) SoC clinical risk model, including Age and serum prostate specific antigen (PSA), (**B**) Engrailed model, (**C**) ExoRNA model and (**D**) ExoGrail model, combining predictors from all three modes of analysis. The full list of variables in each model is available in Table 1. Fill colour shows the risk score distribution of patients with respect to biopsy outcome: No evidence of cancer (Green), Gleason 6 (Blue), Gleason 3 + 4 (Orange), Gleason ≥ 4 + 3 (Red).

As revealed by the distributions of risk scores and AUC, ExoGrail achieved clearer discrimination of disease status Gleason ≥ 3 + 4 disease from other biopsy outcomes when compared to any of the other models (ExoGrail all comparisons *p* < 0.01 bootstrap test, 1000 resamples, Figure 3).

Investigation of risk score distributions found that whilst the SoC model returned respectable AUCs and detection of the higher grade disease (Gleason ≥ 3 + 4), it displayed a relative inability to clearly stratify intermediate disease states. This uncertainty would cause large numbers of patients to be inappropriately selected for further investigation (Figure 3A). For example, to classify 90% of patients with Gleason 7 disease correctly, an SoC risk score of 0.251 would misclassify 64.5% of men with less significant, or no disease. The Engrailed model detailed clearer discrimination, though featured a bimodal distribution of patients without prostate cancer (Figure 3B, green density plot), misidentifying 51.4% of patients with low-grade disease as similar to those with more clinically significant disease (Figure 3B). A similar bimodal distribution was seen for the EN2 plus PSA model (Figure S5). Whilst the AUCs returned for the ExoRNA model were lower, the distribution of risk scores shows that ExoRNA could more accurately discriminate cancer from non-cancer than either the SoC or EN2 models, a key clinical step in the triage of patients prior to biopsy (Figure 3C).

Examination of ExoGrail scores displayed similar distributions for NEC patients as the ExoRNA model whilst also being able to more accurately separate different cancer outcomes from biopsy, resulting in fewer misclassifications of patients without cancer if binary detection of 95% of Gleason ≥ 3 + 4 were considered (28% of NEC patients misclassified). The greater discriminatory ability of the ExoGrail model when biopsy outcomes are considered as a binary Gleason ≥ 3 + 4 threshold can also be seen in Figure S6.

**Figure 4:**
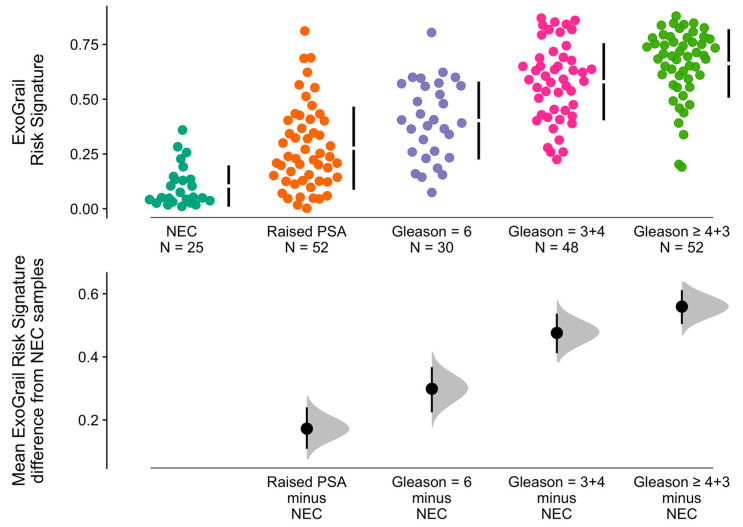
Mean ExoGrail risk score differences between biopsy outcomes, as represented by Cumming estimation plots. Individual patient risk scores (y-axis) are presented as points in the top panel, separated according to Gleason score (x-axis) with gapped vertical lines detailing the mean and standard deviation of each clinical group’s ExoGrail risk score. Mean ExoGrail risk score differences relative to the no evidence of cancer (NEC) group are shown in the bottom panel. Mean difference and 95% confidence intervals are shown as a point estimate and vertical bar, respectively, with density plots generated from 1000 bias-corrected and accelerated bootstrap resamples.

Comparisons of mean ExoGrail scores between groups were performed with resampling and Cumming estimation plots (1000 bias-corrected and accelerated bootstrap resamples, Figure 4). The mean ExoGrail differences between patients with no evidence of cancer on biopsy were: Gleason 6 = 0.3 (95% CI: 0.22–0.37), Gleason 3 + 4 = 0.48 (95% CI: 0.41–0.53) and Gleason ≥ 4 + 3 = 0.56 (95% CI: 0.51–0.61). Of note, patients with no evidence of cancer had a lower ExoGrail risk score (mean difference = 0.17 (95% CI: 0.11–0.24)) than those with a raised PSA but no findings of cancer on biopsy (Figure 4).

**Figure 5:**
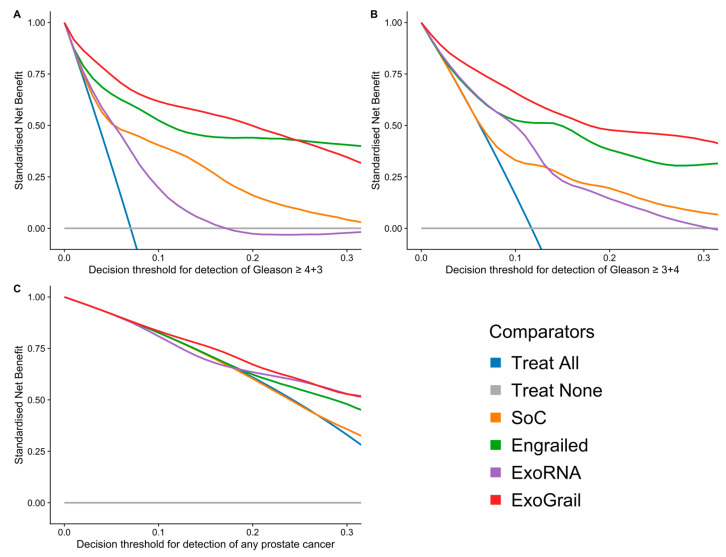
Exploration of the standardised net benefit (sNB) by decision curve analysis (DCA) for adopting risk models to aid the decision to undertake an initial biopsy for patients presenting with a serum PSA ≥ 4 ng/mL, where current clinical practice is to biopsy all patients. The accepted patient/clinician risk threshold for accepting biopsy is detailed on the x-axis. Different biopsy outcomes are shown in each of the three panels; (**A**) detection of Gleason ≥ 4 + 3, (**B**) detection of Gleason ≥ 3 + 4, (**C**) any cancer; Blue—biopsy all patients with a PSA >4 ng/mL, Orange—biopsy patients according to the SoC model, Green—biopsy patients based on the Engrailed model, Purple—biopsy patients based on the exoRNA model, Red—biopsy patients based on a the ExoGrail model. To assess the benefit of adopting these risk models in a clinically relevant population, we used data available from the control arm of the The Cluster Randomized Trial of PSA Testing for Prostate Cancer (CAP) study [42] for proportionally resampling the ExoGrail cohort. DCA curves were calculated from 1000 bootstrap resamples of the available data to match the distribution of disease reported in the CAP trial population. Mean sNB from these resampled DCA results are plotted here. See Methods for full details.

Decision curve analyses examined the net benefit of ExoGrail adoption in a population of patients with a clinical suspicion of prostate cancer and a PSA level suitable to trigger biopsy (≥4 ng/mL). The biopsy of men based upon their ExoGrail risk score provided a net benefit over current standards of care across all decision thresholds examined and was the most consistent amongst all comparator models across a range of clinically relevant endpoints for biopsy (Figure 5).

**Figure 6:**
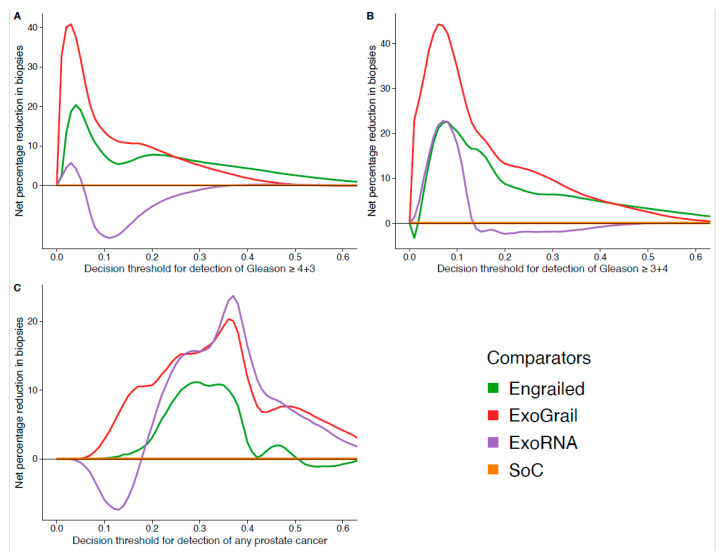
Estimation of biopsy reduction, as calculated by comparing the DCA-calculated net benefit of each risk model to the net benefit of the standard of care (SoC) model. The accepted patient/clinician risk threshold for accepting biopsy is detailed on the x-axis. Different biopsy outcomes are shown in each of the three panels: (**A**) detection of Gleason ≥ 4 + 3, (**B**) detection of Gleason ≥ 3 + 4 and (**C**) any cancer. Coloured lines show differing comparator models; Orange—biopsy patients according to the SoC model, Green—biopsy patients based on the Engrailed model, Purple—biopsy patients based on the ExoRNA model, Red—biopsy patients based on the ExoGrail model. To assess the benefit of adopting these risk models in a clinically relevant population we used data available from the control arm of the CAP study [42] for proportionally resampling the ExoGrail cohort. DCA curves were calculated from 1000 bootstrap resamples of the available data to match the distribution of disease reported in the CAP trial population. Net benefit averaged over all resamples was used to calculate the potential reductions in biopsy rates here. See Methods for full details.

Using the SoC model as the baseline with which to compare the potential for biopsy reduction of each model, we found that ExoGrail could reduce unnecessary biopsy rates by upwards of 40%, depending on accepted patient-clinician risk. For example, if a decision threshold of 0.1 were accepted, representing a perceived risk of 1 in 10 for Gleason ≥ 3 + 4 on biopsy, ExoGrail could result in up to a 35% reduction in unnecessary biopsies of men presenting with a suspicion of prostate cancer, whilst also correctly identifying patients with more aggressive disease. If Gleason ≥ 4 + 3 were considered the threshold of clinical significance, a more conservative decision threshold of 0.05 could save 32% of men from receiving an unnecessary biopsy (Figure 6).

## 4. Discussion

Discriminating disease status in patients before a diagnostic biopsy with higher accuracy than current standards could bring about a sizeable change in treatment pathways and reduce the number of men sent forward for ultimately unnecessary biopsy. Given that up to 75% of patients are negative for prostate cancer when presenting with serum PSA levels ≥ 4 ng/mL [5,43,44], a concentration of research efforts has been made to address this problem. To date, several biomarker panels have been successfully developed to non-invasively detect prostate cancer using urine samples, Gleason ≥ 3 + 4 disease with superior accuracy to current clinically implemented methods, including the PUR model developed by ourselves [20,21,45,46]. However, as only a single aspect of urine, assay method or biological process are assessed by these examples, the heterogeneity of prostate cancer may not be entirely accounted for [47], requiring an approach to be taken that provides a more holistic insight into disease status.

Recent analyses, including those presented here, have demonstrated the added value of integrating multiple prognostic biomarkers within the process of fitting risk models for determining patient risk upon an initial biopsy [23,48]. Urine clearly contains a wealth of useful information concerning the disease status of the prostate through the quantification of cf-RNA transcripts, circulating and cell-free DNA, hypermethylation of DNA, and protein biomarker levels [19,46,49,50,51,52].

Our results show that an improved multivariable risk prediction model can be developed from the careful consideration of information from multiple different urine fractions in men suspected to have prostate cancer. Urinary levels of EN2 protein were quantified by ELISA, whilst the transcript levels of 167 cell-free mRNAs were quantified using NanoString technology. The final model integrating information from those assays with serum PSA levels was deemed ExoGrail. Markers selected for the model include well-known genes associated with prostate cancer and proven in other diagnostic tests, such as *PCA3* [45], *HOXC6* [20], and the *TMPRSS2/ERG* gene fusion [53]. An interaction between urinary EN2 protein levels and quantified transcripts of *SLC12A1* was observed, further demonstrating the benefit of considering information from multiple biological sources (Figure S4).

ExoGrail was able to accurately predict the presence of significant (Gs ≥ 7) prostate cancer on biopsy with an AUC of 0.89, comparing favourably to other published tests (AUCs for Gs ≥ 7: PUR = 0.77 [46], ExoMeth = 0.89 [23], ExoDX Prostate IntelliScore = 0.77 [21], SelectMDX = 0.78 [20], epiCaPture Gs ≥ 4 + 3 AUC = 0.73 [49]). Furthermore, ExoGrail resulted in accurate predictions even when serum PSA levels alone proved inaccurate; patients with a raised PSA but negative biopsy result possessed ExoGrail scores significantly different from both clinically benign patients and those with low-grade Gleason 6 disease, whilst still able to discriminate between more clinically significant Gleason ≥ 7 cancers (Figure 4). The adoption of ExoGrail into current clinical pathways for reducing unnecessary biopsies was considered, showing the potential for up to 32% of patients to safely forgo an invasive biopsy without incurring excessive risk (Figure 6).

ExoGrail was developed with the explicit goal of being robust to potential overfitting and bias, using strong internal validation methods in bootstrap resampling and out-of-bag predictions. Nonetheless, ExoGrail was developed in a relatively small dataset and so requires external validation in an independent cohort before it can be considered for use as a clinical risk model. To this end, we are currently collecting samples from multiple sites in the UK, EU and Canada using an updated ‘At-Home’ Collection system [54]. The At-Home collection system enables biomarker analysis to be performed on urine samples provided by patients at home, which they send in the post to a centralised laboratory. This collection and analysis system will sidestep the need for a visit to the clinic and lead to a postal screening system for prostate cancer diagnosis and prognosis. In this study, we will also assess the potential utility of supplementing MP-MRI with ExoGrail, as MP-MRI can misrepresent disease status, even with rigorous controls in place [6]. The NanoString expression analysis system used in the ExoGrail signature is a rapid and cost-effective analysis system that is also used in the FDA-approved Prosigna Pam50 test for breast cancer aggressiveness [55], making ExoGrail well-positioned for implementation for patient benefit.

## 5. Conclusions

ExoGrail was able to accurately predict the presence of significant (Gs ≥ 7) prostate cancer on biopsy and showed the potential for an important number of patients to safely forgo an invasive biopsy. If validated in future studies, ExoGrail has the potential to positively impact the clinical experience of patients being investigated for prostate cancer that ultimately have no disease or indolent prostate cancer.

## 6. Patents

A patent application has been filed by the authors for the present work and work related to this.

## Figures and Tables

**Table 1:** Characteristics of the ExoGrail development cohort, stratified according to a record of cancer or not, either on an initial biopsy or for a no cancer finding if a biopsy was not performed.

	No Cancer Finding:	Biopsy Positive Cancer Finding
Collection Centre:		
NNUH, n (%)	77 (100)	130 (100)
Age:		
minimum	45.00	53.00
median (IQR)	65.00 (59.00, 71.00)	68.50 (65.00, 76.00)
mean (sd)	65.22 ± 8.10	69.71 ± 7.67
maximum	82.00	91.00
PSA:		
minimum	0.30	4.10
median (IQR)	6.10 (3.70, 8.80)	10.35 (6.82, 16.48)
mean (sd)	7.89 ± 8.72	17.08 ± 18.33
maximum	63.80	95.90
Prostate Size (DRE Estimate):		
Small, n (%)	13 (17)	13 (10)
Medium, n (%)	34 (44)	64 (49)
Large, n (%)	21 (27)	38 (29)
Unknown, n (%)	9 (12)	15 (12)
Gleason Score:		
0, n (%)	77 (100)	0 (0)
6, n (%)	0 (0)	30 (23)
3 + 4, n (%)	0 (0)	48 (37)
4 + 3, n (%)	0 (0)	24 (18)
≥8, n (%)	0 (0)	28 (22)
Biopsy Outcome:		
No Biopsy, n (%)	25 (32)	0 (0)
Biopsy Negative, n (%)	52 (68)	0 (0)
Biopsy Positive, n (%)	0 (0)	130 (100)

**Table 2:** Features positively selected for each model by bootstrap resampling and the Boruta algorithm. Features are selected for each model by being confirmed as important for predicting biopsy outcome, categorised as a modified ordinal variable (see Methods) by Boruta in ≥90% of bootstrap resamples.

	SoC	Engrailed	ExoRNA	ExoGrail
Clinical Parameters	Serum PSA	-	*-*	Serum PSA
	Age	-	*-*	-
ELISA Targets		EN2 (ELISA)	*-*	EN2 (ELISA)
NanoString cf-RNA targets			*ERG* exons *4-5*	*ERG* exons 4-5
			*ERG* exons *6-7*	*ERG* exons 6-7
			*GJB1*	*GJB1*
			*HOXC6*	*HOXC6*
			*HPN*	*HPN*
			*NKAIN1*	*-*
			*PCA3*	*PCA3*
			*PPFIA2*	*PPFIA2*
			*RPLP2*	*-*
			-	*SLC12A1*
			*TMEM45B*	*TMEM45B*
			*TMPRSS2/ERG* fusion	*TMPRSS2/ERG* fusion

**Table 3:** Area under the receiver operator curve (AUC) of all trained models for detecting outcomes of an initial biopsy for varying clinically significant thresholds. Numbers within brackets detail 95% confidence intervals of the AUC, calculated from 1000 stratified bootstrap resamples. Input variables for each model are detailed in Table 1.

Initial Biopsy Outcome:	SoC	Engrailed	ExoRNA	ExoGrail
Gleason ≥ 4 + 3:	0.77 (0.69–0.84)	0.81 (0.74–0.88)	0.67 (0.59–0.75)	0.84 (0.78–0.89)
Gleason ≥ 3 + 4:	0.72 (0.65–0.79)	0.83 (0.77–0.88)	0.77 (0.70–0.83)	0.90 (0.86–0.94)
Any Cancer	0.75 (0.68–0.82)	0.81 (0.74–0.86)	0.81 (0.74–0.87)	0.89 (0.85–0.94)

## Data Availability

All data and code required to reproduce these analyses can be found at https://github.com/UEA-Cancer-Genetics-Lab/ExoGrail_paper.

## Supplementary Materials

The following are available online at https://www.mdpi.com/article/10.3390/cancers13092102/s1, Figure S1: Boruta analysis of variables available for the training of the SoC model, Figure S2: Boruta analysis of variables available for the training of the Engrailed model, Figure S3: Boruta analysis of variables available for the training of the ExoRNA model, Figure S4: Partial dependency plots detailing the marginal effects and interactions of SLC12A1 and urinary EN2 on predicted ExoGrail Risk Score, Figure S5: Risk score distributions of the trained models including the EN2 and PSA model, Figure S6: Density plots detailing risk score distributions generated from four trained models. Table S1: List of all features available for selection as input variables for each model prior to bootstrapped Boruta feature selection, Table S2: AUC of all trained models, including a combination of EN2 and PSA, for detecting outcomes of an initial biopsy for varying clinically significant thresholds. Supplementary Methods.
