# Supplementary material for: Integration of Urinary EN2 Protein & Cell-Free RNA Data in the Development of a Multivariable Risk Model for the Detection of Prostate Cancer Prior to Biopsy
Source: Cancers (Basel). 2021 Apr 27;13(9):2102. doi: 10.3390/cancers13092102 (PMC8123800; doi:10.3390/cancers13092102)
Supplement: Supplementary file 1 [file cancers-13-02102-s001.zip › cancers-1130350 Supplementary Methods.pdf]

*Supplementary Methods*

# Integration of Urinary EN2 Protein & Cell-Free RNA Data in the Development of a Multivariable Risk Model for the Detection of Prostate Cancer Prior to Biopsy

**Shea P. Connell [1], Robert Mills [2], The Movember GAP1 Urine Biomarker Consortium [†], Hardev Pandha [3], Richard Morgan [4], Colin S. Cooper [1], Jeremy Clark [1] and Daniel S. Brewer [1,5,*]**

**Commented [M71]:** Please carefully check the accuracy of names.

**Commented [M72]:** Please kindly consider to move this research group at the end, behind all authors. If you choose to leave it in the middle please add serial number and email.

**Urine collection and processing – The Movember Protocol for Cell-free mRNA RNA extraction**

**Equipment, Reagents and Consumables**

*Chemicals*

- Ethanol AR (VWR International 10107) (100% (vol/vol) COSHH Cat 1 CRITICAL Use fresh solutions or prepare at least weekly. CAUTION! Ethanol is flammable and a contact hazard; wear gloves when handling. Do not ingest.
- Dithiothreitol 1M, (Sigma, 43816-50ML) CAUTION! Toxic if inhaled, swallowed or absorbed through skin. Causes eye irritation. May cause respiratory tract irritation. Use in a chemical fume hood. Wear gloves when handling.
- Glycogen 1ug/ul solution in 2xTE, or GlycoBlue (15 µg/µl, Ambion, Cat no. AM 9516).
- PBS Phosphate buffered saline.
- 500mM EDTA (Sigma)
- Ambion Nuclease-free water – (AM9930, Ambion, Applied Biosystems).

*Plasticware*

- UltraCel-100k Centrifugal Filter unit, 100kDa MWCO (Millipore, UFC910096).
- 1.5 mL non-stick, RNase-free tubes (Ambion, AM12450).
- 2 mL non-stick, RNase-free tubes (Ambion, AM12475).

- 30 mL Syringe (Fisher, HCA-445-005G).
- 7.5 mL Bijou tubes
- RNase-free filter plugged pipette tips for volumes 0.5µl–1000µl (eg Rainin, RT1000F, RT200F, RT20F, RT10F).
- Universal tube, 30 mL (Sterilin).
- 0.8µm filter (Minisart CA Cat. 16592k, Sartorius, or Minisart Cat. FDP-620-070Y, Fisher)

*Kits*

- RNeasy Micro Kit (Qiagen, Cat: 74004, 50 samples): Buffer RLT Harmful by inhalation, in contact with skin and if swallowed. Buffer RW1 Flammable. DNase I Harmful -may cause sensitisation by inhalation and skin contact.
- Nugen Ovation PicoSL WTA2 (Cat. No. 3312-48).
- QIAGEN MinElute Reaction Cleanup Kit (Cat. no. 28204).

*Equipment*

- Fume Hood.
- -80°C freezer.
- Agilent Bioanalyzer
- Large centrifuge (to take 50 mL centrifuge tubes to 3,200g).
- Microcentrifuge.
- Pipettes for volumes 0.5µl – 1000µl (eg Gilson).

*Other*

- Ice, dry ice
- Styrofoam collection box

*Equipment needed in the Clinic.*

- 30 mL screw cap tubes for urine collection.
- Specimen bags for patient to put the bottle of urine into.

- Insulated box with ice for sample storage.
- Bag with absorbent material for sample transport from clinic to the lab.
- 1 mL pipette and tips

*Qiagen Kit notes*

- Add 4 volumes of 100% ethanol to 'Buffer RPE' to obtain a working solution – Freshly Mixing RPE stock with Ethanol each day helps with long term consistency of yield.
- Qiagen RNeasy micro kit (before first use): Prepare 'DNase I' stock solution. Dissolve 550µl of Ambion (Nuclease-free) water 'green lid' by injecting into the vial using a needle and syringe. Divide aliquots for long-term storage, store at -20°C (up to 9 months). Thawed solutions can be stored at 4°C (must be used within 6 weeks).
- Prepare fresh 80% ethanol daily in a capped tube.
- Add 40µl of 1M DTT per mL RLT buffer. DTT-RLT can be stored at room temperature for up to one month).
- Buffer RLT may form a precipitate upon storage. If necessary, redissolve by warming, and then place at room temperature (15–25°C).
- Buffer RLT and Buffer RW1 contain a guanidine salt and are therefore not compatible with disinfecting reagents containing bleach.
- Perform all steps of the procedure at room temperature.
- Perform all centrifugation steps at 20–25°C. Ensure that the centrifuge does not cool below 20°C.
- Air dry tubes for 2 min prior to adding elution water.
- Warming elution water to 45°C can increase yield.
- Extra washes with RLT, RW1 and RPE help decrease false 230 and 280nm wavelength peaks.

**DRE-urine collection for DNA/RNA:**

1. Prepare 30 mL Universal collection bottles, one per patient. Label the collection bottle with patient number, patient name and date.
2. Obtain consent from the patient. Before sample collection the clinician should perform a DRE on the patient's prostate as follows: Apply pressure on the prostate, enough to depress the entire surface of the prostate approximately 1cm, from the base to the apex and from lateral to the median line for each lobe. Perform exactly 3 strokes for each lobe.
3. Ask the patient to provide 'first catch' urine (the first ~30 mL passed) in the Universal sample tube.
4. Place the sample in a Styrofoam box with ice packs in the clinic room. (can use ice, but not ice/water mix as this cools the sample too much causing the urine to go cloudy).
5. Maintain on ice.
6. Within 15 min of sample collection:
7. Invert the DRE urine sample 4 times to resuspend any sediment.
8. Aliquot 4.5 mL of whole urine into capped tubes (3x1 mL, 3x0.5 mL) and freeze at -80°C (or place on dry ice and transfer to the -80°C later).
9. If the total volume of the urine is less than 15 mL then only freeze 3x 0.5 mL.

**Urine Sample processing:**

1. Centrifuge the samples at 1200g for 5min at 6°C. Ensure that the centrifuge brake speed is set on a slow deceleration setting to avoid disruption of the pellet.

1. Carefully and slowly pipette off the supernatant into the 'EV' 30 mL Universal tube. **Place on crushed ice until ready to extract EV RNA - which should be within 4 hours of urine collection.**

**Extracellular Vesicle (EV) Harvest by 100kDa Filter Centrifugation:**

For each sample, label the following with the sample number and an 'X' for EV:

a) 30 mL Syringe

b) Amicon UltraCel -100kDa Centrifugal filter unit (UFC910096) or (#UFC910096, Millipore)

c) 1.5 mL non-stick tube (Ambion AM12450)

d) 30 mL Universal tube

**NB**: Add 40µl of 1M DTT per mL RLT buffer (Qiagen RNeasy Micro kit). DTT-RLT can be stored at room temperature for up to one month).

1. Spin the supernatant at 2000g 5 min r/t.
2. Filter the urine sample to remove any remaining cell debris: Pull the plunger out of a 30 mL syringe and insert the barrel into a 0.8µm filter. Pour the urine into the syringe. Insert the plunger and push the urine through the 0.8um filter.
3. Load the urine supernatant into an UltraCel 100kDa spin-filter unit and centrifuge at 3,400g 10min 21°C.
4. If the urine will not pass through the filter then use a 1 mL pipette to squirt the filter surfaces with the urine and respin 5 min. Repeat until the urine volume is reduced to <500µl. Take care not to touch or damage the filters themselves.
5. Remove the UltraCel 100k unit from the centrifuge and discard flow through. Add the rest of the urine sample from the syringe/filter to the spin unit.
6. Spin the UltraCel 100k unit at 3,400g 10min 21°C until the volume of the sample has reduced to <500µl.
7. Add 15 mL of PBS.
8. Spin at 3,400g 10min 21°C or until the volume is ~200µl.
9. Discard flow through.
10. Pipette out the concentrated sample using a 200µl pipette. Transfer to a 1.5 mL non-stick tube. Measure the volume (Should be 200µl in total). If less, then make up the volume to 200µl with PBS.
11. Immediately rinse the filter unit with 700µl of **RLT/ DTT** buffer from the Qiagen Micro RNeasy kit and add this to the sample tube.

12. Add ethanol to a final concentration of 35%.

    To do this, measure the total vol (ie Sample + RLT). Then multiply this by 0.54 and add this amount of 100% ethanol (usually ~485µl ethanol).

13. Vortex 10-20 sec to mix and disrupt the extracellular vesicles, proceed to RNA extraction with a Qiagen Micro RNeasy kit.

**RNA Extraction from EVs using a Qiagen RNeasy Micro kit.**

**Preparation:**

1. Transfer one RNeasy MiniElute spin column per sample from the fridge the night before and leave at room temperature.
2. Warm the elution water to 45°C.

**For each sample you will need:**

1. An RNeasy MiniElute spin column placed in a 7.5 mL Bijou tube.
2. A 1.5 mL non-stick tube with sample number, date and X (for EV) containing 1µl of 1µg /µl glycogen.
3. 80µl of DNAse 1 mix (10µl of 'DNase I' stock solution with 70µl of 'Buffer RDD'. Mix by inversion).

**Procedure**

1. Place an RNeasy Mini Elute spin column in the neck of a 7.5 mL Bijou collection tube and place that into a large centrifuge - set at 21°C 1500g.
2. Load half of the sample (~700µl) onto the micro filter cartridge.
3. Spin 10-15 sec (or until the mix has passed through the filter - can be up to 1min – the samples that don't spin through the 100kDa unit can cause blockage on the Qiagen column and need longer spinning).
4. Repeat steps 2) and 3).
5. Add 350µl of 'RW1 Buffer' wash, Spin 1500g 10-15 sec.
6. Add 80µl DNAse 1 mix (see above) directly to the membrane of each 'Mini Elute Column'). Leave at room temperature in the centrifuge for 15 min. – can empty the Bijou collection tubes at this point if necessary.
7. Add 350µl **RW1**, spin 15sec.
8. Add 500µl **RPE,** spin 15sec.

9. Add 500µl of freshly diluted 80% ethanol (use the RNAse-free $H_2O$ in the kit) to each 'Mini Elute Column'. Spin 2000g 2 min.
10. Transfer the 'Mini Elute Columns' into new Qiagen collection tubes and place in a microcentrifuge, Spin with the tube lid open at max speed for 5 min. Make sure the tube lid is open as this will aid drying of the filter. Discard flow through and 'collection tube'.
11. Place the 'Mini Elute Column' into a labelled 1.5 mL Ambion non-stick collection tube containing 1ul of 1ug/ul glycogen in 2xTE. Add 20µl of 45°C Qiagen nuclease free water (provided in the kit) to the centre of the membrane.
12. Wait 2-3 min and then centrifuge at max speed for 1 min.
13. Store the RNA sample in a -80°C freezer.

**Notes**: Air drying sample columns for 5 min prior to adding elution water is essential.

Warming elution water to 45°C can increase yield.

Extra washes with RLT, RW1 and RPE help decrease false 230 and 280 nm Spectrophotometer peaks.

Mixing RPE stock with Ethanol on a daily basis helps with long term consistency of yield.

**Amplification of RNA for NanoString analysis.**

Amplify 15-20 ng of EV RNA as quantified by Bioanalyzer.

Use the Nugen Ovation 2 RNA amplification kit as manufacturer's instructions (Nugen Ovation PicoSL WTA2 (3312-48)).

**Clean-up of amplification products**

QIAGEN MinElute Reaction Cleanup Kit (Cat. no. 28204).

1. Aliquot 300 µl of Buffer ERC into a labeled 1.5 mL microcentrifuge tube

2. Add the entire volume (40 µl) of the Nugen Ovation SPIA reaction to the tube.
3. Vortex for 5 sec, then spin briefly (5 sec) in a microcentrifuge.
4. Label a Min Elute spin column and place in a collection tube.
5. Load the sample/buffer mixture onto the column.
6. Centrifuge for 1 min at 13,000 g in a microcentrifuge.
7. Discard the flow-through and replace the column in the same collection tube.
8. Add 750 µl of Buffer PE to the column.
9. Centrifuge for 1 min at maximum speed.
10. Discard the flow-through and replace the column in the same collection tube.
11. Centrifuge the column for an additional 2 min at maximum speed to remove all residual Buffer PE. Note: Residual ethanol from the wash buffer will not be completely removed unless the flow-through is discarded before this additional centrifugation.
12. Discard the flow-through with the collection tube. Blot the column onto clean, absorbent paper to remove any residual wash buffer from the tip of the column. Note: Blotting the column tip prior to transferring it to a clean tube is necessary to prevent any wash buffer transferring to the eluted sample.
13. Place the column into a clean, labelled 1.5 mL microcentrifuge tube.
14. Add 20 µl of room temperature, Nuclease-free Water (green: D1) from the NuGEN® kit to the centre of each column. Note: Ensure that the water is dispensed directly onto the membrane for complete elution of the bound cDNA.
15. Let the column stand for 1 min at room temperature.
16. Centrifuge for 1 min at maximum speed.
17. Discard the column and measure the volume recovered.
18. Mix the sample by vortexing, then spin briefly.
19. Add 1/10th vol of 1xTE and store at -80°C.

**Supplementary References**


1. Christensen, R. H. B. *ordinal—Regression Models for Ordinal Data*. (2018).
2. Brown, M. *rmda: Risk Model Decision Analysis*. (2017).
3. Robin, X.; *Turck, N.; Hainard, A.; Tiberti, N.; Lisacek, F.;* Sanchez, J-C.; Müller, M. pROC: an open-source package for R and S+ to analyze and compare ROC curves. *BMC Bioinformatics* **12,** 77 (2011).
4. Miranda, K. C.; Bond, D.T.; McKee, M.; Skog, J.; Paunescu. T.G.; Da Silva, N.; BRown, D.; Russo, L.M. Nucleic acids within urinary exosomes/microvesicles are potential biomarkers for renal disease. *Kidney Int.* **78,** 191–199 (2010).
