# Supplementary material for: Integration of Urinary EN2 Protein & Cell-Free RNA Data in the Development of a Multivariable Risk Model for the Detection of Prostate Cancer Prior to Biopsy
Source: Cancers (Basel). 2021 Apr 27;13(9):2102. doi: 10.3390/cancers13092102 (PMC8123800; doi:10.3390/cancers13092102)
Supplement: Supplementary file 1 [file cancers-13-02102-s001.zip › cancers-1130350 supplementary.pdf]

Supplementary Materials

Integration of Urinary EN2 Protein & Cell-Free RNA Data in the Development of a Multivariable Risk Model for the Detection of Prostate Cancer Prior to Biopsy

Shea P. Connell [1], Robert Mills [2], Hardev Pandha [3], Richard Morgan [4], Colin S. Cooper [1], Jeremy Clark [1] and Daniel S. Brewer [1,5,*]; The Movember GAP1 Urine Biomarker Consortium [†]

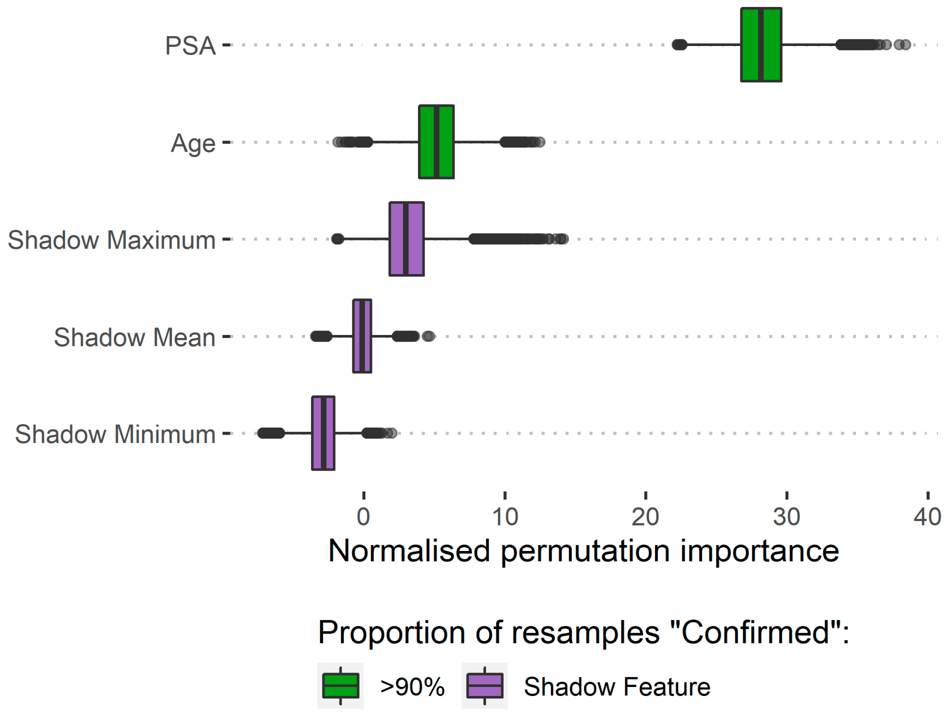

Figure S1. Boruta analysis of variables available for the training of the SoC model. Variable importance was determined over 1,000 bootstrap resamples of the available data and the decision reached recorded at each resample. Colour indicates the proportion of the 1,000 resamples a variable was confirmed to be important in. Variables confirmed in at least 90% of resamples were selected for training predictive models (Green).

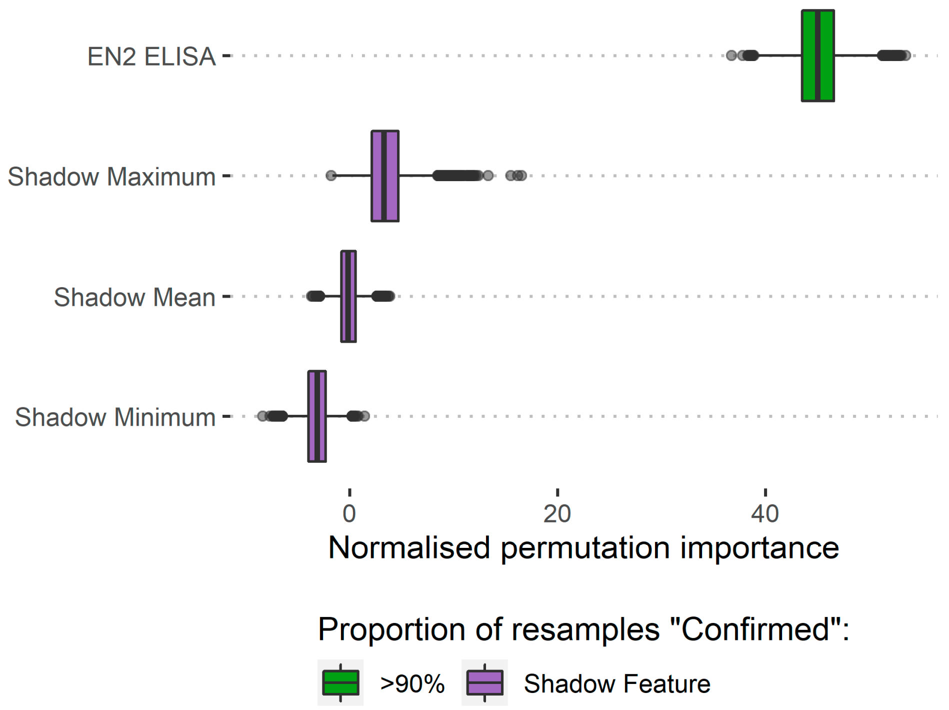

Figure S2. Boruta analysis of variables available for the training of the Engrailed model. Variable importance was determined over 1,000 bootstrap resamples of the available data and the decision reached recorded at each resample. Colour indicates the proportion of the 1,000 resamples a variable was confirmed to be important in. Variables confirmed in at least 90% of resamples were selected for training predictive models (Green).

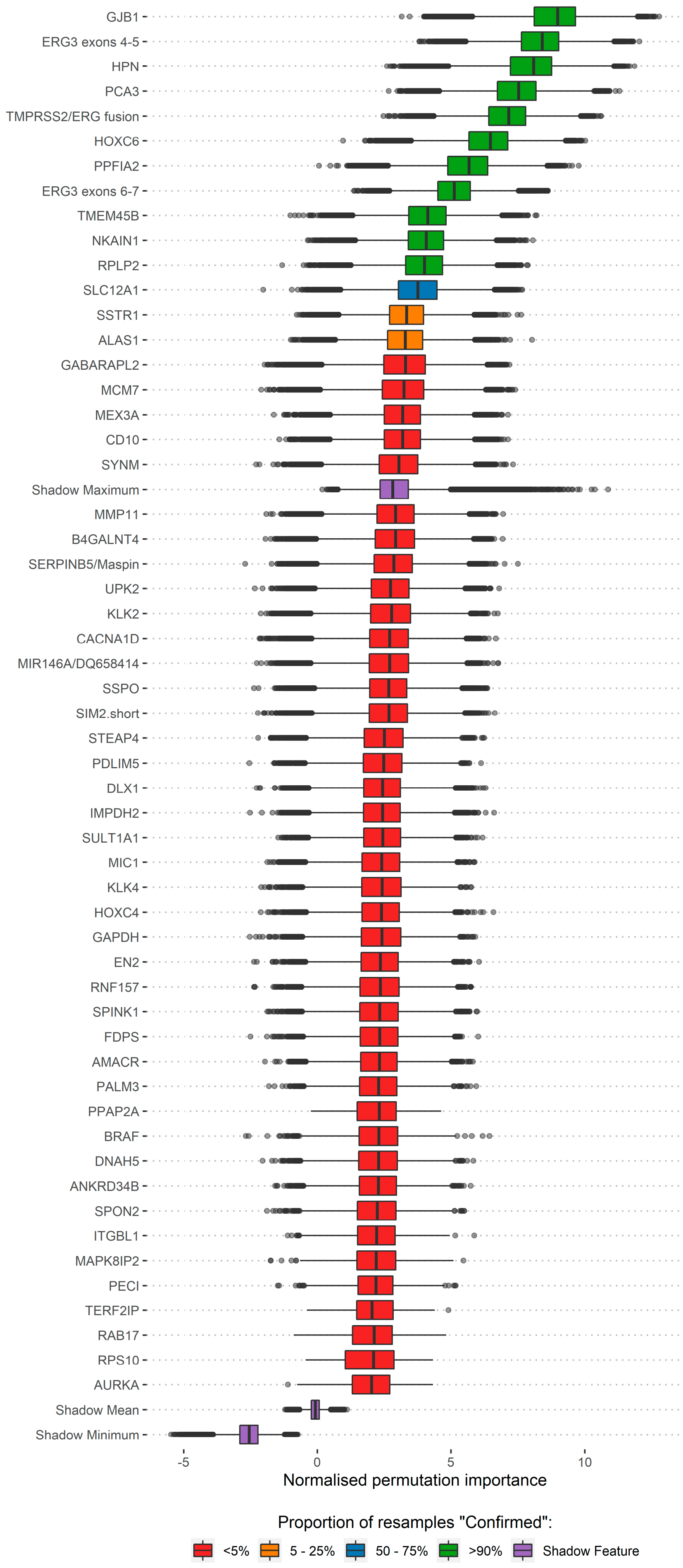

GJB1
ERG3 exons 4-5
HPN
PCA3
TMPRSS2/ERG fusion
HOXC6
PPFIA2
ERG3 exons 6-7
TMEM45B
NKAIN1
RPLP2
SLC12A1
SSTR1
ALAS1
GABARAPL2
MCM7
MEX3A
CD10
SYNM
Shadow Maximum
MMP11
B4GALNT4
SERPINB5/Maspin
UPK2
KLK2
CACNA1D
MIR146A/DQ658414
SSPO
SIM2.short
STEAP4
PDLIM5
DLX1
IMPDH2
SULT1A1
MIC1
KLK4
HOXC4
GAPDH
EN2
RNF157
SPINK1
FDPS
AMACR
PALM3
PPAP2A
BRAF
DNAH5
ANKRD34B
SPON2
ITGBL1
MAPK8IP2
PECI
TERF2IP
RAB17
RPS10
AURKA
Shadow Mean
Shadow Minimum
-5
0
5
10
Normalised permutation importance
Proportion of resamples "Confirmed":
<5%
5 - 25%
50 - 75%
>90%
Shadow Feature

Figure S3. Boruta analysis of variables available for the training of the ExoRNA model. Variable importance was determined over 1,000 bootstrap resamples of the available data and the decision reached recorded at each resample. Colour indicates the proportion of the 1,000 resamples a variable was confirmed to be important in. Variables confirmed in at least 90% of resamples were selected for training predictive models. Those variables rejected in every single resample are not shown here, but the full list of inputs for the ExoRNA model can be seen in Table S1.

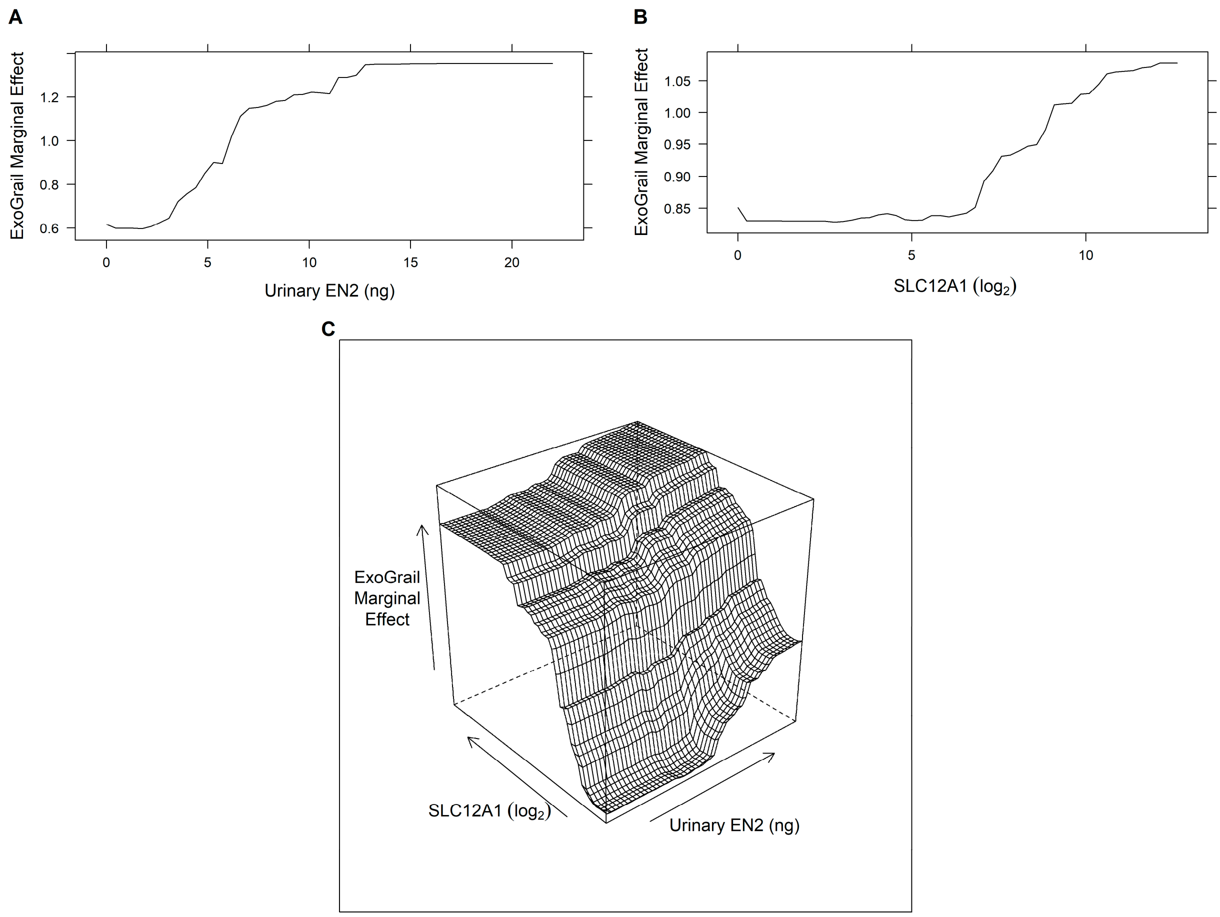

Figure S4 Partial dependency plots detailing the marginal effects and interactions of SLC12A1 and urinary EN2 on predicted ExoGrail Risk Score. **A** - Partial dependency of ExoGrail on urinary EN2, **B** - Partial dependency of ExoGrail on SLC12A1, **C** - Partial dependency of ExoGrail on both SLC12A1 and urinary EN2.

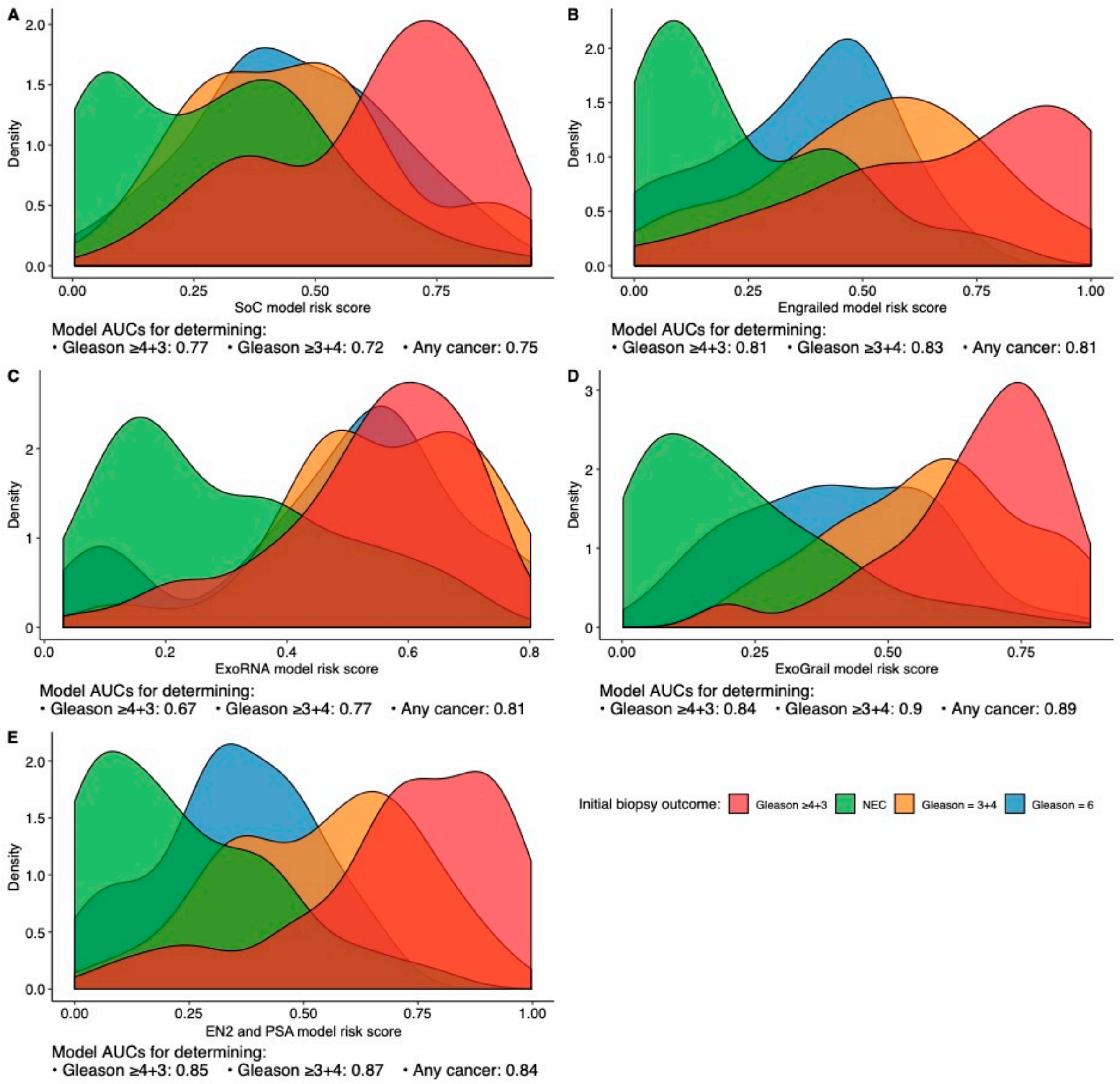

Figure S5. Risk score distributions of the trained models including the EN2 and PSA model, calculated as the out-of-bag predictions and represented as density plots. AUCs for each model's predictive ability for clinically relevant outcomes are detailed underneath each panel. Each random forest model was fit using differing input variables; A - SoC clinical risk model, including Age and PSA, B - Engrailed model, C -ExoRNA model and D - ExoGrail model, combining predictors from all three modes of analysis E -EN2 and PSA model. The full list of variables in each model is available in Table 1. Fill colour shows the risk score distribution of patients with respect to biopsy outcome: No evidence of cancer (Green), Gleason 6 (Blue), Gleason 3+4 (Orange), Gleason ≥ 4+3 (Red). Both the Engrailed model and the EN2&PSA model featured a bimodal risk score distribution in samples from men with no evidence of prostate cancer (NEC) (green density plots), falsely identifying some patients with NEC and low-grade disease as similar to those with more clinically significant G3+4 disease (orange density plots). The bimodal distribution of NEC is not a feature of the ExoGrail model and separation is improved. In the EN2+PSA model a bimodal distribution is now seen in Gleason 3+4 patients (E, orange density plot), falsely identifying some patients with high grade disease as similar to those with lower grade disease. Finally, the ExoGrail model also appears to improve delineation of the G≥4+3 patients manifesting as a more distinct peak in the red density plot of D compared to EN2 and EN2&PSA plots.

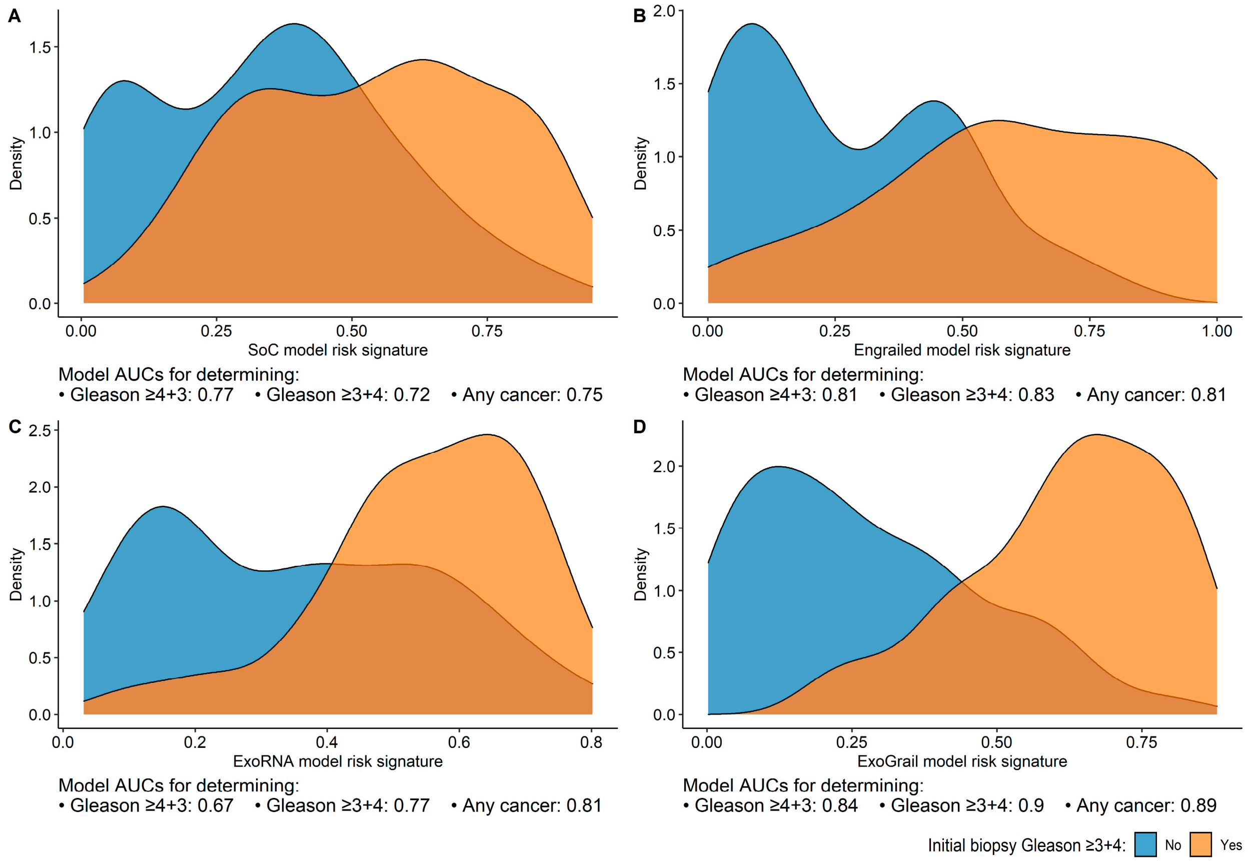

Figure S6. Density plots detailing risk score distributions generated from four trained models. Models A to D were trained with different input variables; **A** - SoC clinical risk model, including Age and PSA, **B** - Engrailed model, **C** -ExoRNA model and **D** - ExoGrail model, combining the predictors from all three previous models. The full list of variables in each model is available in Table 1. Fill colour shows the risk score distribution of patients with a significant biopsy outcome of Gs ≥ 3+4 (Orange) or Gs ≤ 6 (Blue)

Table S1. List of all features available for selection as input variables for each model prior to bootstrapped Boruta feature selection.

SoC	Engrailed	ExoRNA	ExoGrail
PSA	EN2 ELISA	March5	PSA
UrineVol		AATF	UrineVol
DRESize		ABCB9	DRESize
Age		ACTR5	Age
		AGR2	EN2 ELISA
		ALAS1	March5
		AMACR	AATF
		AMH	ABCB9
		ANKRD34B	ACTR5
		ANPEP	AGR2
		APOC1	ALAS1
		ARexon9	AMACR
		ARexons4.8	AMH
		ARHGEF25	ANKRD34B
		AURKA	ANPEP
		B2M	APOC1
		B4GALNT4	ARexon9
		BRAF	ARexons4.8
		BTG2	ARHGEF25
		CACNA1D	AURKA
		CADPS	B2M
		CAMK2N2	B4GALNT4
		CAMKK2	BRAF
		CASKIN1	BTG2
		CCDC88B	CACNA1D
		CD10	CADPS
		CDC20	CAMK2N2
		CDC37L1	CAMKK2
		CDKN3	CASKIN1

CKAP2L	CCDC88B
CLIC2	CD10
CLU	CDC20
COL10A1	CDC37L1
COL9A2	CDKN3
CP	CKAP2L
CTA.211A9.5.MIATNB	CLIC2
DLX1	CLU
DNAH5	COL10A1
DPP4	COL9A2
EIF2D	CP
EN2	CTA.211A9.5.MIATNB
ERG3.exons.4.5	DLX1
ERG3.exons.6.7	DNAH5
ERG5	DPP4
FDPS	EIF2D
FOLH1.PSMA.NAALAD1	EN2
GABARAPL2	ERG3.exons.4.5
GAPDH	ERG3.exons.6.7
GCNT1	ERG5
GJB1	FDPS
GOLM1	FOLH1.PSMA.NAALAD1
HIST1H1C	GABARAPL2
HIST1H1E	GAPDH
HIST1H2BF	GCNT1
HIST1H2BG	GJB1
HIST32HA	GOLM1
HMBS	HIST1H1C
HOXC4	HIST1H1E
HOXC6	HIST1H2BF
HPN	HIST1H2BG

HPRT	HIST32HA
IFT57	HMBS
IGFBP3	HOXC4
IMPDH2	HOXC6
ISX	HPN
ITGBL1	HPRT
ITPR1	IFT57
KLK2	IGFBP3
KLK3.PSA.exons1.2	IMPDH2
KLK3.PSA.exons2.3	ISX
KLK4	ITGBL1
LASS1	ITPR1
LBH	KLK2
MAK	KLK3.PSA.exons1.2
MAPK8IP2	KLK3.PSA.exons2.3
MCM7	KLK4
MCTP1	LASS1
MDK	LBH
MED4	MAK
MEMO1	MAPK8IP2
Met	MCM7
MEX3A	MCTP1
MFSD2A	MDK
MGAT5B	MED4
MIC1	MEMO1
MIR146A.DQ658414	Met
MIR4435.1HG.lOC541471	MEX3A
MKi67	MFSD2A
MMP11	MGAT5B
MMP25	MIC1
MMP26	MIR146A.DQ658414

MNX1	MIR4435.1HG.lOC541471
MSMB	MKi67
MXI1	MMP11
MYOF	MMP25
NAALADL2	MMP26
NEAT1	MNX1
NKAIN1	MSMB
NLRP3	MXI1
OGT	MYOF
OR52A2.PSGR	NAALADL2
PALM3	NEAT1
PCA3	NKAIN1
PCSK6	NLRP3
PDLIM5	OGT
PECI	OR52A2.PSGR
PPAP2A	PALM3
PPFIA2	PCA3
PPP1R12B	PCSK6
PSTPIP1	PDLIM5
PTN	PECI
PTPRC	PPAP2A
PVT1	PPFIA2
RAB17	PPP1R12B
RIOK3	PSTPIP1
RNF157	PTN
RP11.244H18.1.P712P	PTPRC
RP11.97O12.7	PVT1
RPL18A	RAB17
RPL23AP53	RIOK3
RPLP2	RNF157
RPS10	RP11.244H18.1.P712P

RPS11	RP11.97O12.7
SACM1L	RPL18A
SChLAP1	RPL23AP53
SEC61A1	RPLP2
SERPINB5.Maspin	RPS10
SFRP4	RPS11
SIM2.long	SACM1L
SIM2.short	SChLAP1
SIRT1	SEC61A1
SLC12A1	SERPINB5.Maspin
SLC43A1	SFRP4
SLC4A1.S	SIM2.long
SMAP1.ex.7.8	SIM2.short
SMIM1	SIRT1
SNCA	SLC12A1
SNORA20	SLC43A1
SPINK1	SLC4A1.S
SPON2	SMAP1.ex.7.8
SRSF3	SMIM1
SSPO	SNCA
SSTR1	SNORA20
ST6GALNAC1	SPINK1
STEAP2	SPON2
STEAP4	SRSF3
STOM	SSPO
SULF2	SSTR1
SULT1A1	ST6GALNAC1
SYNM	STEAP2
TBP	STEAP4
TDRD	STOM
TERF2IP	SULF2

TERT	SULT1A1
TFDP1	SYNM
Timp4	TBP
TMCC2	TDRD
TMEM45B	TERF2IP
TMEM47	TERT
TMEM86A	TFDP1
TMPRSS2.ERG.fusion	Timp4
TRPM4	TMCC2
TWIST1	TMEM45B
UPK2	TMEM47
VAX2	TMEM86A
VPS13A	TMPRSS2.ERG.fusion
ZNF577	TRPM4
	TWIST1
	UPK2
	VAX2
	VPS13A
	ZNF577

Table S2. AUC of all trained models, including a combination of EN2 and PSA, for detecting outcomes of an initial biopsy for varying clinically significant thresholds. Brackets detail 95% confidence intervals of the AUC, calculated from 1,000 stratified bootstrap resamples.

Initial biopsy outcome:	SoC	Engrailed	ExoRNA	ExoGrail	EN2_PSA
Gleason ≥4+3:	0.77 (0.69 - 0.84)	0.81 (0.73 - 0.88)	0.67 (0.60 - 0.75)	0.84 (0.78 - 0.89)	0.85 (0.78 - 0.91)
Gleason ≥3+4:	0.72 (0.65 - 0.79)	0.83 (0.77 - 0.88)	0.77 (0.70 - 0.83)	0.90 (0.85 - 0.94)	0.87 (0.82 - 0.91)
Any Cancer	0.75 (0.68 - 0.82)	0.81 (0.75 - 0.86)	0.81 (0.75 - 0.87)	0.89 (0.85 - 0.93)	0.84 (0.78 - 0.89)
